# Utilising the COM-B model to interpret barriers and facilitators to cervical cancer screening in young women

**DOI:** 10.1177/13591053241281405

**Published:** 2024-09-28

**Authors:** Sonia Shpendi, Paul Norman, Jilly Gibson-Miller, Rebecca K. Webster

**Affiliations:** University of Sheffield, UK

**Keywords:** cancer, cervical cancer, cervical screening, health behaviour, health psychology, pap smear, sexual health, women’s health

## Abstract

As most women now reaching the age for cervical cancer screening (24.5 years old) in the UK will be HPV vaccinated, their current perspectives on screening can inform effective interventions to increase screening uptake (and thus, early detection). Twenty-four interviews were conducted with women aged 24–30 years old to explore their views on cervical cancer screening (*n* = 12 attendees and *n* = 12 non-attendees). Reflexive thematic analysis generated six themes that were then mapped onto the COM-B model. Reflective motivations (e.g. reassurance) were key facilitators to screening attendance for both groups. Social opportunities (e.g. open communication) contrasted between the groups, with attendees more likely to have discussed screening with friends. Automatic motivations (e.g. embarrassment) were key barriers to attending screening in both groups. Notably, HPV vaccination did not factor into the decision to attend screening. Interventions to increase screening uptake may target motivational and social factors.

## Introduction

Cervical cancer remains a public health concern worldwide and in the United Kingdom (UK), despite the availability of free effective preventative measures such as cervical screening under the National Health Service (NHS, 2023). According to the European age-standardised incidence rate, cancer rates in the UK have increased by 13% in young females over the last decade (Cancer Research UK, 2022). Furthermore, epidemiological studies have revealed the highest prevalence of cervical cancer cases in situ, preinvasive lesions that without treatment may develop into invasive carcinoma, is in females aged 25–29 (Cancer Research UK, 2019). Therefore, cervical screening programmes remain central to early diagnosis, with 99.8% of cervical cancer cases being preventable (Roope, 2021).

However, there is a clear age gradient in cervical screening uptake in the UK with younger women (59.7% of 25–29-year-olds) being less likely to attend than older women (72.1% of 45–49-year-olds) (NHS Digital, 2021). One reason for this may be that young women tend to report more practical barriers to screening attendance and lower perceptions of risk for developing cancer (Waller et al., 2009). Therefore, it is important to understand the unique perspectives and barriers to screening in this age group. A recent systematic review highlighted the utility of the COM-B model in conceptualising barriers and facilitators reported in previous studies of the uptake of cervical screening in young women (Shpendi et al., 2024). The COM-B model is designed to provide an overarching framework that captures all factors that influence behaviour change (Michie et al., 2011). The COM-B model states that behaviour is the outcome of (1) physical and psychological *capability* to perform the behaviour; (2) physical and social *opportunity* to do so; and (3) reflective (conscious thought and decision-making) and automatic (habits and subconscious processes) *motivation* (Michie et al., 2011). The COM-B model has previously been used to examine cervical screening behaviours as well as to illustrate barriers to attendance among different age groups (Alam et al., 2021; O’Donovan et al., 2021). Shpendi et al. (2024) reported that key barriers to attending cervical screening identified among those aged 30 and younger aligned with the physical opportunity (e.g. low accessibility and financial constraints) and automatic motivation (e.g. embarrassment) components of the COM-B model. In contrast, psychological capability (e.g. knowledge) and social opportunity (e.g. communication with friends and family) were commonly cited factors facilitating screening attendance.

It is important to note that the majority of women in the UK who now reach the screening age (24.5 years old) will have received the Human Papillomavirus (HPV) vaccination (UK Health Security Agency, 2022). Persistent HPV infection remains one of the most common causes of cervical cancer (Bedell et al., 2020), therefore, it follows that a reduction in HPV infection via vaccination would reduce the prevalence of cervical cancer. This means that vaccination could potentially influence cervical screening uptake both positively and negatively. On the one hand, it might be that lower attendance could result from a reduced perceived need for cervical screening among the vaccinated. Alternatively, vaccination could serve to alleviate anxieties or provide reassurance surrounding the likelihood of receiving positive screening results and therefore increase attendance. Indeed, previous studies have reported that HPV vaccination is positively related to the uptake of cervical cancer screening (Beer et al., 2014; Boone et al., 2016; Kitchener et al., 2018). Shpendi et al. (2024) also highlighted that young women who were HPV vaccinated were more likely to have attended screening compared to those not vaccinated. It is therefore important to explore further how being vaccinated encourages screening uptake through in-depth qualitative research.

Women now reaching the screening age for cervical cancer are some of the first cohorts of women in the UK to have had the opportunity to receive the HPV vaccination. Research has not yet reported on the views of screening in this group of women. Insights from qualitative research would allow for a greater understanding of the impact of the vaccination on screening uptake for cervical cancer, whilst adding a more in-depth perspective to previous findings that have investigated factors that are most prominent for screening attendance. The current study aims to explore, via semi-structured interviews, the barriers and facilitators, including HPV vaccination, to attending cervical screening in women aged 24–30 years old who have (attenders) versus those who have not (non-attenders) attended their first cervical screening invitation. We aimed to gain a richer and more in-depth understanding of the decision-making process surrounding screening, which may contribute to refining initiatives to promote cervical screening.

## Method

### Participants

Participants were a convenience sample of young women eligible for screening based in the UK, recruited from online platforms (e.g. Twitter, Call for Participants and LinkedIn). Eligible participants were aged 24–30 years old, to recruit those who had reached the eligibility age of first invitation to the NHS cervical screening programme in the UK (24.5 years old), but also to allow for delayed uptake of screening. Those who had attended a cervical cancer screening under the age of 24 years (before eligibility for the NHS screening programme), had a history of cervical cancer and/or had a hysterectomy procedure were not eligible to participate. Potential participants were first invited to complete a screening questionnaire that consisted of four questions to determine eligibility for the interview study (i.e. age, geographical location, whether they had been screened previously under the age of 24, and hysterectomy history) as well as one question on whether or not they had attended their first cervical cancer screening to categorise participants into groups. Eligible participants were then contacted via email in the order they completed the screening questionnaire with further information about the interview study and available interview slots.

Braun and Clarke (2021a) have questioned the relevance of the concept of data saturation, as well as the practice of predetermining a required sample size, for reflexive thematic analysis. Instead, they argue that determining a sample size is a pragmatic exercise that should be decided in situ (i.e. during data collection) and informed by the extent to which the data are adequate, in terms of their depth, richness and/or complexity, to address the research question. In the present study, it was decided to initially interview 12 attendees and 12 non-attendees, before deciding whether or not to interview further participants.

Online interviews were organised with those expressing an interest to participate until the initial recruitment quotas had been met (i.e. 12 participants per group). Participants were provided with a unique participant ID, a link to an information sheet and a consent form, to be completed before the interview. After completion of the interview, participants were sent an online shopping voucher via email (£20 Amazon voucher). The study received ethics approval from the University of Sheffield Research Ethics Committee.

In total, 24 participants were interviewed, comprising 12 attendees and 12 non-attendees (see Table 1 for sample characteristics). The median age of the final sample was 27 (range = 26-29), all participants identified as women, and the majority were either partnered (*n* = 11) or married (*n* = 4). The majority of participants were HPV vaccinated (*n* = 13) and had received two or more doses (*n* = 8) (see Table 2).

**Table 1.** Demographic characteristics of participants (*N* = 24).

Characteristics	Median (range)	Attendees *n* (%)	Non-attendees *n* (%)	Total *n* (%)
Age	27 (25–30)			
Ethnicity				
White British or White Other		9 (75)	7 (58)	16 (67)
Asian or Asian British		1 (8.)	4 (33)	5 (21)
Black, Black British, Caribbean or African		2 (17)	1 (8)	3 (13)
Place of residence				
London		4 (33)	7 (58)	11 (46)
Yorkshire and The Humber		1 (8)	2 (17)	3 (13)
East of England		0 (0)	2 (17)	2 (8)
West Midlands (England)		2 (17)	0 (0)	2 (8)
East Midlands (England)		2 (17)	0 (0)	2 (8)
Southeast (England)		2 (17)	0 (0)	2 (8)
Southwest (England)		1 (8)	1 (8)	2 (8)
Education				
Undergraduate degree		4 (33)	6 (50)	10 (42)
Postgraduate degree		5 (42)	4 (33)	9 (38)
Higher certificates		3 (25)	0 (0)	3 (13)
A-level, national diploma or equivalent		0 (0)	1 (8)	1 (4)
PhD		0 (0)	1 (8)	1 (4)
Relationship status				
Partnered		7 (58)	4 (33)	11 (46)
Single		2 (17)	5 (42)	7 (29)
Married		3 (25)	1 (8)	4 (17)
Other		0 (0)	2 (17)	2 (8)
Religion				
Not religious		7 (58)	5 (42)	12 (50)
Catholicism/Christianity		3 (25)	4 (33)	7 (29)
Islam		2 (17)	2 (17)	4 (17)
Sikhism		0 (0)	1 (8)	1 (4)
Employment status				
Employed		8 (67)	7 (58)	15 (63)
Part-time		1 (8)	5 (42)	6 (25)
Studying		2 (17)	0 (0)	2 (8)
Self-employed/Freelance		1 (8)	0 (0)	1 (4)

**Table 2.** HPV vaccination data of participants (*N* = 24).

HPV vaccination	Attendees *n* (%)	Non-attendees *n* (%)	Total *n* (%)
HPV vaccinated			
Yes	7 (58)	6 (50)	13 (54)
No	5 (42)	6 (50)	11 (46)
Number of doses			
Two or more	4 (33)	4 (33)	8 (33)
One	3 (25)	2 (17)	5 (21)
Location of vaccination			
School	5 (42)	4 (33)	9 (38)
Healthcare services	2 (17)	2 (17)	4 (17)

### Interviews

Twenty-four in-depth online interviews were conducted (12 attendees and 12 non-attendees), between August and September 2022 by SS. Semi-structured interviews encouraged an open discussion and allowed participants to express themselves freely. Core questions focused on screening experiences, perceptions and attitudes towards screening, and knowledge and experiences of the HPV vaccination. The topic guide gave the researcher a flexible set of questions and prompts on relevant topics, allowing participants’ views and experiences to influence the discussion (see Supplemental Materials). SS regularly reflected in a diary on her subjective role within the interview process and analysis as a young woman within the eligibility age for this study, having attended her first cervical cancer screening and with experience of the HPV vaccine. SS completed data collection and analysis from a critical realist position (Alderson, 2021) which recognises screening as a universal ‘procedure’ to which women have complex and varied experiences constructed through social phenomena and influence. Interviews were audio recorded and transcribed verbatim. All participants provided consent and completed a demographic questionnaire obtaining data about age, ethnicity, place of residence, education, relationship status, religion and employment status. Interviews lasted a median of 28 minutes (16–45 minutes) and were carried out by SS.

### Analysis

Reflexive thematic analysis (RTA) (Braun and Clarke, 2021b; Braun et al., 2023) enables flexible analysis and, most importantly, allows for analysing data inductively and deductively. RTA has been employed to explore people’s experiences of a range of health issues (e.g. Bell et al., 2024; Clarkson et al, 2023; Plunkett and Pilkington, 2024). An important feature of RTA is that the researcher reflects on their role in knowledge construction throughout the research process. SS kept a reflexive diary during interviews and data analysis to draw on during analysis. This reflective practice served as a reminder of the objectives of the study and a way to acknowledge the researchers’ biases and assumptions about the subject matter as a woman who closely identifies with the participants in the study.

RTA utilises both semantic codes and themes, referring to explicit and surface meanings of data and latent analysis that considers underlying ideas and/or patterns in the data. In the present study, data analysis comprised two stages. In stage 1 initial themes were generated using inductive reasoning, whereas in stage 2 deductive reasoning was used to map the themes onto COM-B model components. A similar approach was followed by Moore et al. (2023) in their RTA of the impact of endometriosis on quality of life in which they used an inductive approach to identify initial themes, followed by a deductive approach to map the themes onto pre-established illness representation dimensions. Analysis for attendees and non-attendees was conducted separately, and then combined. Data familiarisation began for SS by listening to the transcripts and transcribing the recordings verbatim. During stage 1, transcripts were coded by SS, who generated initial themes using NVivo14 software. SS referred back to her reflective diary entries to refresh interview memory and recall initial observations, for example, participants’ tone of voice and emotional cues when discussing points. No coding book was used, as RTA discourages coding books (Braun and Clarke, 2024). Questions that arose with the team during discussions and presentation opportunities at the time of analysis aided the development and review of initial themes. SS also used mindmaps to visualise codes and initial themes when building the narrative of the findings and finalising the themes. In stage 2, the COM-B model was used to interpret the themes according to how they reflected the psychological constructs represented in the model and agreed by all authors.

## Results

**Figure 1.**
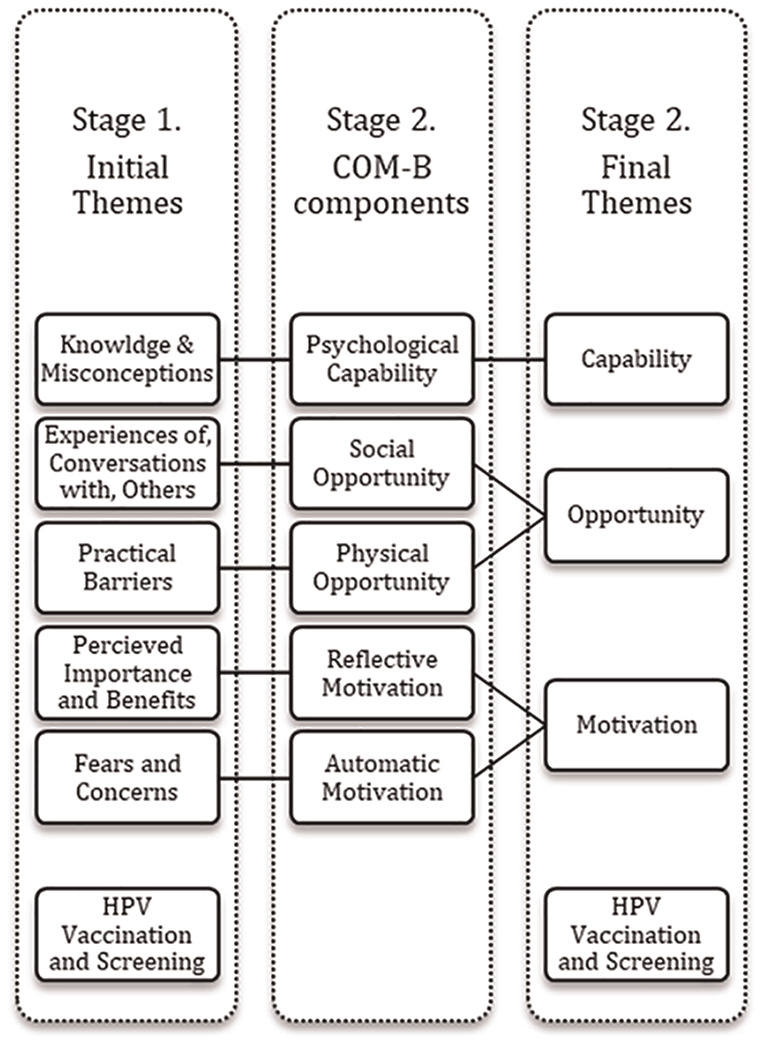
Visual representation of themes and two-stage analysis.

Figure 1 presents the six themes of barriers and facilitators to screening that were identified in Stage 1 of the analysis. In stage 2, five of these themes were then mapped onto COM-B model components (i.e. capability, opportunity, motivation), which left the remaining theme focusing on HPV vaccination, presented as a separate concept.

### Stage 1: Inductive analysis

#### Knowledge and misconceptions

Unsurprisingly, attendees expressed more knowledge surrounding screening and the procedure than non-attendees, which may influence the uptake of screening. This was not only due to experiencing screening first hand but also to more information-seeking behaviours, such as online searching and research about screening. The non-attendee group were aware of the initial age of eligibility for screening and were expectant of the invitation but lacked knowledge of the procedure itself. Similar to attendees, non-attendees expressed a desire to research screening further but only some actively had done so.

I’m not very familiar with the topic yet. And I wanted to do some research and understand what it’s about and what it entails. So, once I have the understanding then I would make the decision of whether to go or not. (P20, non-attendee)

Misconceptions surrounding the age of screening were one of the most prominent barriers to uptake amongst non-attenders. Participants expressed screening as something done when older and that they felt ‘too young’ to attend, often referring to 30 years old and older, or relative to mothers and aunties.

Yeah, I don’t think I can take it too seriously because I feel like I am just still young. (P19, non-attendee)

Most of the participants felt that improving education and awareness around the topic was the best way to improve knowledge about screening and the procedure. This was consistent across both groups, with school education as a common recommendation and a focus on specific aspects of what screening is and why it is done, to improve uptake. This also included the possibility of adding more information to the invitation and from GP/health worker settings, as well as hearing from other community members about their experiences.

If I hadn’t of been able to see the video or had sort of like people that I know, talked to me about the experience, or even had, like STI tests, like they were in a similar I’ve done this similar way. uhm I think I would have probably put it off like most people. (P5, attendee)

Attendees shared coping strategies and recommendations for easing the screening experience. Further knowledge surrounding various options to personalise screening was also highlighted as one way to improve confidence in screening and overall experiences. For example, one participant emphasised ‘distracting yourself’ with music and maintaining personal hygiene to be more relaxed, whereas a few participants suggested bringing a friend or family member for moral support.

Maybe kind of making it more clear to women or people have like uhm cervixes like what their kind of not rights what their kind of what they’re entitled to in the procedure. (P10, attendee)

The nature of cervical cancer was also highlighted as a facilitator to screening, as women were aware that cervical cancer can be asymptomatic, but treatable if diagnosed early.

#### Experiences of, and conversations with, others

Several attendees had experience and/or knowledge of a close family member’s experience with cancer. Specific family histories of breast cancer, cervical cancer and ovarian cancer were shared by some, in which two of these cases were maternal figures. For some participants, maternal encouragement was a driver in attending their screening, as well as having friends and family working within the health industry.

My mom is a nurse. So, she’s always very hot on [me] attending these sorts of things. (P4, attendee)

In attendees, overall conversations and open communication with friends and family about cervical screening and similar topics were common. Contrary to this, non-attendees highlighted a lack of conversation with friends and family, resulting in unawareness of those who had attended the screening or not in their close circles.

I’ve got friends that have had issues, we’re very open me and my friends about kind of, you know, all sorts like periods we like talk about it all. We’re very open. (P4, attendee)

Not really just like, it’s like occasionally mentioned. Like, it’s not like a topic of conversation or something. (P18, non-attendee)

I haven’t met anyone who has gone to get this screening. So, I don’t know who to ask or like, does it hurt? Like, I don’t know. (P14, non-attendee)

Negative experiences and stories were shared by friends and family with participants in both groups. However, the influence of this was more prominent amongst non-attendees. In the few instances where negative stories were shared, this worked to deter participants from screening. One participant who had missed an appointment due to illness had not rebooked due to negative stories shared via friends.

Very uncomfortable they didn’t feel well after and I think it just put me off as well cos I’m the sort of person that’s quite squeamish about things anyway. (P23, non-attendee)

Non-attendees also expressed negative perceptions of, and previous interactions with, healthcare providers. In general, non-attendees did not express the same level of trust in healthcare services as attendees. Furthermore, attendees also frequently highlighted the role of the nurse/health worker during screening and the importance of friendliness and reassurance.

They don’t care, they don’t care about how we feel. And so then that makes things like this, is it going to be a horrible experience? … I think that’s that kind of general feeling that I have towards it. (P22, non-attendee)

I think the best way to put it is that I trust the NHS. The NHS has always done right by me. (P9, attendee)

It must be essential that the nurses you know, nice and calm and as I said, friendly, has a smile wants to be there. (P4, attendee)

Irrespective of screening uptake, both groups indicated that there was a lack of strong presence of screening topics on social media, but both felt this could be an opportunity to further spread awareness. However, the emphasis remained on encouragement and information coming from trustworthy sources, such as GPs and healthcare professionals, instead of social media.

#### Practical barriers

Busy lifestyles were reported in both groups, but in particular amongst non-attendees when discussing the reasons for non-attendance. Specifically, this often referred to changing routines and work-life balance. Emphasis on the specific timing of invitations and personal availability at the time of reminders was also reported in both groups. Many attendees reported being caught at a ‘good time’, whereas non-attendees highlighted the importance of timing for them in aiding uptake.

When I received the letter, I was quite, there wasn’t many things on my mind, my schedule was fully empty. It wasn’t a busy time of my life. (P6, attendee)

I’m gonna start working. So you never, you never really know when you’re going to be available. (P16, non-attendee)

A key issue for non-attendees was also negative perceptions and difficulties getting an appointment. They often referred to assumptions that GP booking services are inconvenient and tedious, based on experiences of booking general appointments with GP services. Two participants explicitly stated that booking an appointment was the main reason for not yet having attended screening. However, the suggestion of pre-booked appointments as a solution yielded mixed responses; one participant felt this would help with hesitation in booking, whereas two others did not feel this would be convenient and rather increased pressure to attend.

Because I’m assuming that it’s not that I don’t want to, I’d love to, I think the main problem is that you call them they’ll probably say, well, we don’t have any availability this week, or next week, or in a month, you can come in like six months, or something. (P16, non-attendee)

That’s what puts me off because I know how hard it is to like speak to a- like get an appointment like speak to anyone at like the appointments desk. (P18, non-attendee)

##### Perceived importance and benefits

Screening was frequently assumed as ‘important’, without often providing reasoning for this assertion. Most commonly participants reported attending for ‘reassurance’, particularly through the ‘knowledge’ and ‘confidence’ in one’s health. Consideration of the costs and benefits of screening was common with one participant stating that, for them, ‘the advantages outweigh the disadvantages’ (P7, attendee).

Similarly, around half of those in the non-attendee group also reported reassurance as a main reason why they would intend to screen in the future, as well as sharing the viewpoint that screening is important. The majority of participants in the non-attendee group also indicated an intention to screen.

The top three reasons so I would say my health and reassurance … uhm so yeah reassurance that everything’s all right. (P4, attendee)

Something like this is quite good because you getting you know, the reassurance and everything’s normal. (P19, non-attendee)

The most common reason for non-attendance among participants was a lack of priority setting when it came to booking and/or attending an appointment. Some examples of this were due to lower perceived risk amongst some in the non-attendee group, with a lack of symptoms and a low number of sexual partners as reasons for not attending the screening.

Not actually booked in or did anything about it, because I thought, well, it’s not really a priority right now. (P13, non-attendee)

#### Fears and concerns

Participants across both groups expressed emotions related to embarrassment, fear and nerves when discussing screening. However, for non-attendee participants, this was cited as a barrier to screening and a reason for non-attendance. Although both groups expressed similar emotions, a clear difference was evident in the non-attendees’ confidence in their ability to cope with screening and overcome these emotions and complete screening. The invasiveness of the procedure and the intimacy of the area seemed to ‘*put off*’ participants from attending.

Also a big part is just putting it off because I really didn’t like the sound of having to do it – it’s quite invasive. (P24, non-attendee)

Difficult if it entails more than a regular visit to a gynaecologist and I’m already not happy with that. It’s one of those. That’s why I’m like, No, not for me. But I know it’s necessary but at the same time I’m scared. (P14, non-attendee)

Fear or concerns about the result of the screening were mentioned in both groups but did not seem to impact uptake in attendees (perhaps because participants also felt that screening was important exactly because it could detect cervical cancer early). However, a lack of knowledge regarding results and details of what positive or negative results meant was apparent across both groups.

I definitely worry about results and stuff. (P23, non-attendee)

For attendees, the preventative nature and opportunity to attend screening were also reported, with emphasis on the test being free. Similarly, non-attendees also mentioned this as a benefit to the screening process.

I mean what would be more uncomfortable if I don’t get it done and then there is like a problem later down the line it’s better to kind of go and get it caught early if there is anything. (P11, attendee)

#### HPV vaccination and screening

Low awareness of the vaccination and the link with screening was evident across both groups; therefore, there was no indication that the HPV vaccination played an active role in the decision-making process for participants’ screening behaviour.

I’ve got the vaccine I feel like I didn’t even really think about the fact that I’d had the vaccine when I went to screening. (P11, attendee)

So I guess it’s because I haven’t really heard much about it. Again, I haven’t thought about it until you asked. (P16, non-attendee)

Reactions to the effectiveness of the vaccination were also positive across all participants. Most participants were also aware that the vaccination was not fully protective, and that screening would still be needed.

Well, I will still go. It’s only 90% [effective against cervical cancer], there’s 10% left. (P4, attendee)

However, information about the vaccine’s effectiveness also seemed to increase intentions to attend screening by assuring protection whilst also lessening concerns about screening outcomes, indicating a possible facilitating role of vaccination in screening.

On the other hand, one unvaccinated non-attendee also expressed more motivation to attend screening knowing that they did not have the vaccination. However, after discussing the HPV vaccination, some non-attendee participants did feel that being vaccinated meant they could ‘put [screening] to a later date’ (P19, non-attendee) or be screened less regularly (P14, non-attendee). Some non-vaccinated participants from both groups also expressed more interest in taking the vaccine than attending screening.

‘It wouldn’t discourage me from screening from going and get in, and still having the smear test and everything. (P5, attendee)

If I could be offered a vaccine at this stage? I would do it. And I would also get the courage to go and get the screening over and done with. (P15, non-attendee)

I think actually hearing that as well would probably make me more positive about going for my screening as well. (P23, non-attendee)

### Stage 2: Deductive analysis

The COM-B model (Michie et al., 2011) was used to provide an overarching theoretical framework for interpreting the initial themes identified in stage 1 of the data analysis, to represent the nature of the factors influencing screening uptake in behavioural terms. In the discussion between the authors, the themes were mapped onto COM-B model components as shown in Figure 1. We employed deductive reasoning to map themes to COM-B model components by analysing the extent to which each theme represented each concept. For example, the theme ‘Knowledge and Misconceptions’ illustrated how knowledge influenced screening uptake and was therefore mapped onto ‘Psychological Capability’, which refers to how an individual’s capability leads to action. We were able to map each theme to each component of the COM-B model, except for ‘Physical Capability’ which was not discussed in our data. Further, it was not possible to map the theme ‘HPV Vaccination and Screening’ onto a COM-B model component and, as a result, it remained as a fourth, separate theme.

## Discussion

The present study used the COM-B model of behaviour change to interpret barriers and facilitators to cervical cancer screening among young women, focusing on first screening experiences and invitations. Inductive RTA identified six themes that were then mapped onto three COM-B model components, with an additional theme unique to this data set. Thus, we have conceptualised factors that influence screening uptake in attendees and non-attendees, into four final themes: Capability, Opportunity, Motivation and HPV Vaccination and Screening. Psychological capability varied between groups, with attendees sharing more knowledge about screening and the procedure, while age-related misconceptions were prominent amongst non-attendees. Differences in social opportunities, such as open communication with friends and family, were some of the key differences cited between attendees and non-attendees. Greater reflective motivation, such as considering screening as ‘important’ and a source of ‘reassurance’, were important facilitators for both groups. In contrast, automatic motivation, particularly feelings of embarrassment, acted as a barrier for both groups. Finally, participants’ decision to attend cervical screening or not did not appear to be influenced by HPV vaccination status.

Environmental contexts, specifically social influences, were one of the most widely cited factors that impacted screening behaviour and differentiated attendees from non-attendees. Attendees reported that open conversations with family and friends facilitated normalising and increasing screening awareness in this age group. However, these open conversations were not exclusive to screening but also extended to sexual and women’s health topics in general, which aided in normalising the discussion of cervical screening. In contrast, lack of conversation and negative peer influence were cited multiple times by non-attendees, and although not explicitly reported as a barrier, could impact other factors such as knowledge and awareness of screening overall. However, participants who did not engage in conversation surrounding these topics recognised the benefit and sought more opportunities to discuss with friends and family (Coleman et al., 2007; Leahey et al., 2011).

Limited knowledge and awareness of screening remain an issue with this age group, particularly among those who hadn’t attended screening. Specifically, gaps in knowledge regarding the necessity for screening and the procedure were most prominent. Educational interventions could target specific age-related beliefs in this group, such as misconceptions about being ‘too young’ for cervical screening. This misconception could also impact young women’s perceived risk of cervical cancer, outweighing the known benefits and importance of screening. Educational-based interventions may need to target both psychological capability and reflective motivation to be effective. Furthermore, healthcare professionals were a desired source of information and encouragement regarding cervical screening, as well as members of the community when discussing personal experiences. Although social media could be an avenue to explore for spreading awareness in younger generations (Plackett et al., 2020), this age group may also benefit from reminders and prompts from their GP services to reinforce the importance of screening to increase psychological capability.

Motivational processes, both reflective and automatic were salient in both groups. Consistent with previous research (Shpendi et al., 2024), emotional responses to screening (e.g. embarrassment, fear and nerves) were consistently raised by both attendees and non-attendees as were concerns about the test result. It is striking that while both groups mentioned similar barriers, for many attendees, these concerns were offset by the potential benefits and the importance attached to screening. In addition, attendees reported employing various coping strategies, such as breathing and listening to music, to deal with fears and worries about attending screening. Highlighting such coping strategies in pre-screening information could benefit non-attendees. Interestingly, almost all non-attendees in the current study reported an intention to screen in the future. This may reflect an ‘intention-behaviour gap’ (Sheeran and Webb, 2016) such that positive intentions to attend cervical screening may not always translate into actual attendance at screening. Although intentions to screen are more prevalent in younger women when compared to older women (Waller et al., 2012), younger women are less likely to attend than older women; for example, attendance rates are only 59.7% for 25–29-year-olds in England, compared with 72.1% for 45–49-year-olds (NHS Digital, 2021). This suggests that other factors, including those identified in the current study (e.g. embarrassment and worries) may prevent many young women from acting on their positive intentions to attend cervical screening. Tailored interventions that target the commonly perceived barriers for this age group are likely to be required to increase uptake. Given that prior attendance at cervical screening is a strong predictor of continued attendance (Taylor-Phillips et al., 2013; Labeit and Peinemann, 2015), it is imperative that young women are encouraged and supported to attend their first cervical screening appointment. Encouragingly, previous research suggests that interventions may be more successful among hesitant groups that show an intention to screen than among resistant groups with no intention to screen (Betsch et al., 2015). Although long-term non-attendance cannot be determined at the first invitation, targeting this group could be most effective as, if successful, this is likely to translate into continued attendance given that past behaviour is one of the strongest predictors of future behaviour (McEachan et al., 2011).

Furthermore, as previously reported, healthcare providers also play a crucial role in screening uptake and experiences (O’Connor et al., 2014). Negative interactions and practical barriers to accessing healthcare services were noted among non-attendees in the current study, leading to further fears of being ‘dismissed’ or ‘not taken seriously’. Healthcare providers could use their position positively to influence screening uptake in young women. Specifically, healthcare providers were considered the most desired source of information and encouragement when it came to screening. A desire for a female nurse or healthcare provider was prominent, as well as options to further ‘customise’ the experience to aid young women during screening. For example, some participants weren’t aware they could choose to be accompanied by someone to the appointments and during if desired, highlighting a further lack of knowledge regarding screening options.

An important finding in this study emphasised the role of participants’ attitudes and perspectives on the HPV vaccination on cervical screening attendance. Participants stated that the effectiveness of the HPV vaccination would increase their confidence in attending screening, and news of the vaccine’s effectiveness was well received. Perhaps contrary to expectations, a novel finding of this study is that participants reported that they did not necessarily consider their vaccination status in their decision to screen, indicating that vaccination status may not be predictive of screening uptake. Improving HPV vaccination knowledge and awareness may boost screening uptake, however, by providing young women with education on the benefits of the vaccination, whilst alleviating stressors linked with cervical screening results and emphasising the advantages of being vaccinated. This highlights a possible avenue to utilise the HPV vaccination as a facilitator in future interventions to promote screening.

The current study had a number of strengths. Most notably, the use of semi-structured interviews allowed for flexibility to explore in-depth, women’s views on cervical screening. Furthermore, the RTA enabled a two-stage analysis process whereby inductive reasoning generated themes that gave insight into barriers and facilitators to screening, whilst a second deductive analysis stage allowed us to map these themes onto a theoretical model of behaviour change, the COM-B model. It should be noted that the principal researchers’ position within RTA may have influenced data collection and analysis, given their demographic and previous research on the topic. However, this was managed throughout by active reflection. Reflexive practice supports the flexible, yet systematic approach to thematic analysis and is considered a strength of this method, which values the researcher’s subjectivity and in-depth interaction with the data as the primary tool in discerning meaning from data (Braun and Clarke, 2021b). Reflective notes also illustrated the benefits of the researchers’ demographic in helping to create rapport with participants during interviews. Given the sensitivity of the topic and personal experiences being shared, discussing this with someone of a similar age and gender can help create a more open and understanding environment during interviews.

Another key strength of the study is that it was carried out at a unique period during which some of the first HPV vaccination cohorts became eligible for screening under the NHS (24.5 years old and older). This allowed for discussion of HPV vaccination and screening with a sample of first-invitation attendees and non-attendees. However, previous qualitative research has suggested that participants are not always consciously aware of the ‘real’ reason for screening attendance or non-attendance (Waller et al., 2009), therefore it is common to see similar explanations and emotions shared. For example, negative attributes of screening (e.g. embarrassment) are frequently used as justification for non-attendance; however, these attributes are also equally cited by attendees. Future quantitative research might validate the present findings by assessing the prevalence of these barriers and facilitators and the strength of their relationships with screening behaviour in a larger sample of young women.

## Conclusion

Cervical cancer screening behaviour is complex and presents unique challenges in ensuring young women are adequately informed and encouraged to attend. Our findings suggest that more prompts and accurate information are needed from healthcare professionals, as well as encouragement to engender open discussions amongst peers. Furthermore, addressing factors that may be hindering those with positive intentions to attend cervical screening, such as feelings of embarrassment regarding the procedure, is crucial to increasing the uptake of cervical screening in young women. Future work could also examine further the impact of the HPV vaccination and how this could be utilised to promote attendance at cervical screening.

## Supplemental Material

sj-docx-1-hpq-10.1177_13591053241281405 – Supplemental material for Utilising the COM-B model to interpret barriers and facilitators to cervical cancer screening in young women Supplemental material, sj-docx-1-hpq-10.1177_13591053241281405 for Utilising the COM-B model to interpret barriers and facilitators to cervical cancer screening in young women by Sonia Shpendi, Paul Norman, Jilly Gibson-Miller and Rebecca K. Webster in Journal of Health Psychology

## References

[bibr1-13591053241281405] Alam Z Hanjani S Dean L , et al. (2021) Cervical cancer screening among immigrant women residing in Australia: A systematic review. Asia-Pacific Journal of Public Health 33(8): 816–827. 33829888 10.1177/10105395211006600

[bibr2-13591053241281405] Alderson P (2021) Critical Realism for Health and Illness Research: A Practical Introduction. Bristol: Policy Press.

[bibr3-13591053241281405] Bedell SL Goldstein LS Goldstein AR , et al. (2020) Cervical cancer screening: Past, present, and future. Sexual Medicine Reviews 8(1): 28–37. 31791846 10.1016/j.sxmr.2019.09.005

[bibr4-13591053241281405] Beer H Hibbitts S Brophy S , et al. (2014) Does the HPV vaccination programme have implications for cervical screening programmes in the UK? Vaccine 32(16): 1828–1833. 24530938 10.1016/j.vaccine.2014.01.087 PMC3991313

[bibr5-13591053241281405] Bell BT Norminton S Dollimore K. (2024) ‘I’ve learned a lot about myself this year’: Young student women’s perceptions of their cumulative use of digital fitness technologies across the Covid-19 pandemic. Journal of Health Psychology 29(9): 1046–1058. DOI: 10.1177/13591053231225598. 38279803 PMC11301956

[bibr6-13591053241281405] Betsch C Korn L Holtmann C (2015) Don’t try to convert the antivaccinators, instead target the fence-sitters. Proceedings of the National Academy of Sciences 112(49): E6725–E6726. DOI: 10.1073/pnas.1516350112. PMC4679061 26598650

[bibr7-13591053241281405] Boone SD Pinkston CM Baumgartner KB , et al., (2016) Associations between prior HPV4 vaccine doses and cervical cancer screening participation. Cancer Epidemiology 42: 108–114. 27100836 10.1016/j.canep.2016.04.003

[bibr8-13591053241281405] Braun V Clarke V (2021a) To saturate or not to saturate? Questioning data saturation as a useful concept for thematic analysis and sample-size rationales. Qualitative Research in Sport Exercise and Health 13(2): 201–216.

[bibr9-13591053241281405] Braun V Clarke V (2021b) Thematic Analysis: A Practical Guide. London: Sage.

[bibr10-13591053241281405] Braun V Clarke V (2024) Supporting best practice in reflexive thematic analysis reporting in Palliative Medicine: A review of published research and introduction to the Reflexive Thematic Analysis Reporting Guidelines (RTARG). Palliative Medicine 38(6): 608–616. 38469804 10.1177/02692163241234800 PMC11157981

[bibr11-13591053241281405] Braun V Clarke V Hayfield N , et al. (2023) Doing reflexive thematic analysis. In: Bager-Charleson S McBeath A (eds) Supporting Research in Counselling and Psychotherapy: Qualitative, Quantitative, and Mixed Methods Research. London: Palgrave Mcmillan, pp. 19–38.

[bibr12-13591053241281405] Cancer Research UK (2019) State of the Nation April 2019 - Cancer Research UK. Available at: https://www.cancerresearchuk.org/sites/default/files/state_of_the_nation_april_2019.pdf (accessed 6 December 2023).

[bibr13-13591053241281405] Cancer Research UK (2022) Young People’s Cancer Istatistics. Available at: https://www.cancerresearchuk.org/health-professional/cancer-statistics/young-people-cancers/incidence#ref-2 (accessed 6 December 2023).

[bibr14-13591053241281405] Clarkson C Scott HR Hegarty S , et al. (2023) ‘You get looked at like you’re failing’: A reflexive thematic analysis of experiences of mental health and wellbeing support for NHS staff. Journal of Health Psychology 28(9): 818–831. 36597919 10.1177/13591053221140255 PMC10387714

[bibr15-13591053241281405] Coleman L Cox L Roker D (2007) Girls and young women’s participation in physical activity: Psychological and social influences. Health Education Research 23(4): 633–647. 17897930 10.1093/her/cym040

[bibr16-13591053241281405] Kitchener H Gittins M Cruickshank M , et al. (2018) A cluster randomized trial of strategies to increase uptake amongst young women invited for their first cervical screen: The STRATEGIC trial. Journal of Medical Screening 25(2): 88–98. 28530513 10.1177/0969141317696518 PMC5956569

[bibr17-13591053241281405] Labeit A Peinemann F . (2015) Breast and cervical cancer screening in Great Britain: Dynamic interrelated processes. Health Economics Review 5(1): 32. 26487452 10.1186/s13561-015-0065-3 PMC4615931

[bibr18-13591053241281405] Leahey TM LaRose JG Fava JL , et al. (2011) Social influences are associated with BMI and weight loss intentions in young adults. Obesity 19(6): 1157–1162. 21164501 10.1038/oby.2010.301 PMC3079776

[bibr19-13591053241281405] McEachan RRC Conner M Taylor NJ , et al. (2011) Prospective prediction of health-related behaviours with the theory of planned behaviour: A meta-analysis. Health Psychology Review 5(2): 97–144.

[bibr20-13591053241281405] Michie S Van Stralen MM West R (2011) The behaviour change wheel: A new method for characterising and designing behaviour change interventions. Implementation Science 6: 1–12. 21513547 10.1186/1748-5908-6-42 PMC3096582

[bibr21-13591053241281405] Moore C Cogan N Williams L (2023). A qualitative investigation into the role of illness perceptions in endometriosis-related quality of life. Journal of Health Psychology 28(12): 1157–1171. 37358039 10.1177/13591053231183230 PMC10571435

[bibr22-13591053241281405] NHS (2023) Cervical Screening. Available at: https://www.nhs.uk/conditions/cervical-screening/ (accessed 6 December 2023).

[bibr23-13591053241281405] NHS Digital (2021) Cervical Screening Programme England 2020-21. Available at: http://digital.nhs.uk/pubs/cervical2021 (accessed 30 January 2024).

[bibr24-13591053241281405] O’Connor M Murphy J Martin C , et al. (2014) Motivators for women to attend cervical screening: The influential role of GPs. Family Practice 31(4): 475–482. 24927724 10.1093/fampra/cmu029

[bibr25-13591053241281405] O’Donovan B Mooney T Rimmer B , et al. (2021) Advancing understanding of influences on cervical screening (non)-participation among younger and older women: A qualitative study using the theoretical domains framework and the COM-B model. Health Expectations 24(6): 2023–2035. 34476875 10.1111/hex.13346 PMC8628586

[bibr26-13591053241281405] Plackett R Kaushal A Kassianos A , et al. (2020) Use of social media to promote cancer screening and early diagnosis: Scoping review. Journal of Medical Internet Research 22(11): e21582. 10.2196/21582 PMC7683249 33164907

[bibr27-13591053241281405] Plunkett C Pilkington M (2024) Beliefs, screening attitudes and breast cancer awareness of young women with neurofibromatosis type 1: A reflexive thematic analysis. Journal of Health Psychology 30(3): 369–383. 38859614 10.1177/13591053241255053 PMC11894900

[bibr28-13591053241281405] Roope R (2021) Cervical cancer: A unique approach for a unique disease. Royal College of General Practitioners. Available at: https://www.gov.uk/government/statistics/human-papillomavirus-hpv-vaccine-coverage-estimates%0A (accessed 6 December 2023).

[bibr29-13591053241281405] Sheeran P Webb TL (2016) The intention–behavior gap. Social and Personality Psychology Compass 10(9): 503–518.

[bibr30-13591053241281405] Shpendi S Gibson-Miller J Norman P , et al. (2024) Identifying the key barriers, facilitators and factors associated with cervical cancer screening attendance in young women: A systematic review. University of Sheffield. [Unpublished] 10.1177/17455057251324309 PMC11907612 40080394

[bibr31-13591053241281405] Taylor-Phillips S O’Sullivan E Kearins O , et al. (2013) The effects of a UK review of breast cancer screening on uptake: An observational before/after study. Journal of Medical Screening 20(2): 86–90. 24009089 10.1177/0969141313497198 PMC3807969

[bibr32-13591053241281405] UK Health Security Agency (2022) Human Papillomavirus (HPV) Vaccine Coverage Estimates in England: 2020 to 2021. Available at: https://assets.publishing.service.gov.uk/media/63a5b83be90e07048f4d598f/hpr1322-HPV2.pdf (accessed 6 December 2023).

[bibr33-13591053241281405] Waller J Bartoszek M Marlow L , et al. (2009) Barriers to cervical cancer screening attendance in England: A population-based survey. Journal of Medical Screening 16(4): 199–204. 20054095 10.1258/jms.2009.009073

[bibr34-13591053241281405] Waller J Jackowska M Marlow L , et al. (2012) Exploring age differences in reasons for nonattendance for cervical screening: A qualitative study. BJOG 119(1): 26–32. 21668764 10.1111/j.1471-0528.2011.03030.x

