# Supplementary material for: Utilising the COM-B model to interpret barriers and facilitators to cervical cancer screening in young women
Source: J Health Psychol. 2024 Sep 28;30(9):2340–53. doi: 10.1177/13591053241281405 (PMC12322326; doi:10.1177/13591053241281405)
Supplement: sj-docx-1-hpq-10.1177_13591053241281405 – Supplemental material for Utilising the COM-B model to interpret barriers and facilitators to cervical cancer screening in young women [file sj-docx-1-hpq-10.1177_13591053241281405.docx]

**Interview schedule**

Main questions:

1. What do you know about cervical cancer screening?
2. Have you attended your screening?
3. What are some of the reasons you think why you haven’t yet attended your screening?

*Prompt: What is it about that that stops you from going do you think?*

*Prompt: What is it about that that you think you can overcome and still attend in comparison to other reasons?*

1. What are some of the reasons you think why you took the steps to attend your screening?

*Prompt: How do you think you were able to overcome your personal barriers to screening?*

*Prompt: How was your experience?*

*Prompt: What expectations did you have about screening before attending?*

*How did you feel after?*

1. What would help you to attend your screening?

*Alternative: what do you think would help others to attend their screening?*

1. Overall how do you feel about cervical cancer screening?

*Prompt: Have you ever spoken to friends or family about screening?*

*Media and social media?*

*Top few reasons why you go?*

1. Are you aware of the HPV vaccination and what it is for?
   - 1. What made you decide to get it?

*Prompt:* Have you heard of any recent studies or news reports about the effectiveness of the HPV vaccine?

If not…a recent study using the population-based cancer registry showed a 90% effectiveness against cervical cancer and a steep decline in cervical cancer cases.

How do you feel about that?

Did you think of vaccines when screening?

1. Has Covid changed your views?
